# The CCTG PA.7 phase II trial of gemcitabine and nab-paclitaxel with or without durvalumab and tremelimumab as initial therapy in metastatic pancreatic ductal adenocarcinoma

**DOI:** 10.1038/s41467-022-32591-8

**Published:** 2022-08-26

**Authors:** Daniel J. Renouf, Jonathan M. Loree, Jennifer J. Knox, James T. Topham, Petr Kavan, Derek Jonker, Stephen Welch, Felix Couture, Frederic Lemay, Mustapha Tehfe, Mohammed Harb, Nathalie Aucoin, Yoo-Joung Ko, Patricia A. Tang, Ravi Ramjeesingh, Brandon M. Meyers, Christina A. Kim, Pan Du, Shidong Jia, David F. Schaeffer, Sharlene Gill, Dongsheng Tu, Chris J O’Callaghan

**Affiliations:** 1 Pancreas Centre BC, Vancouver, BC Canada; 2 Division of Medical Oncology, BC Cancer, Vancouver, BC Canada; 3 Princess Margaret Cancer Centre, UHN, University of Toronto, Toronto, ON Canada; 4 Department of Medicine and Oncology, Sir Mortimer B. Davis Jewish General Hospital, Segal Cancer Centre, McGill University, Montreal, QC Canada; 5 The Ottawa Hospital, University of Ottawa, Ottawa, ON Canada; 6 London Regional Cancer Program, London, ON Canada; 7 CHU de Quebec Research Centre and Faculty of Medicine, Laval University, Quebec City, QC Canada; 8 Faculty of Medicine and Health Sciences, University of Sherbrooke, Sherbrooke, QC Canada; 9 Hematology and Medical Oncology Division, Centre Hospitalier Universitaire de Montreal, University of Montreal, Montreal, QC Canada; 10 The Moncton City Hospital, Moncton, NB Canada; 11 Hopital Cite-de-la-Sante, Laval, QC Canada; 12 Department of Medicine, Sunnybrook Odette Cancer Centre, Toronto, ON Canada; 13 University of Calgary, Calgary, AB Canada; 14 Nova Scotia Cancer Centre and Dalhousie University, Halifax, NS Canada; 15 Juravinski Cancer Centre, Hamilton, ON Canada; 16 CancerCare Manitoba, Winnipeg, MB Canada; 17 Predicine, Inc., Hayward, CA USA; 18 Department of Pathology and Laboratory Medicine, University of British Columbia, Vancouver, BC Canada; 19 Division of Anatomic Pathology, Vancouver General Hospital, Vancouver, BC Canada; 20 Canadian Cancer Trials Group, Queen’s University, Kingston, ON Canada

**Keywords:** Pancreatic cancer, Genetics research, Cancer genomics, Translational research

## Abstract

Immunotherapy-based monotherapy treatment in metastatic pancreatic ductal adenocarcinoma (mPDAC) has shown limited benefit outside of the mismatch repair deficiency setting, while safety and efficacy of combining dual-checkpoint inhibitor immunotherapy with chemotherapy remains uncertain. Here, we present results from the CCTG PA.7 study (NCT02879318), a randomized phase II trial comparing gemcitabine and nab-paclitaxel with and without immune checkpoint inhibitors durvalumab and tremelimumab in 180 patients with mPDAC. The primary endpoint was overall survival. Secondary endpoints included progression-free survival and objective response rate. Results of the trial were negative as combination immunotherapy did not improve survival among the unselected patient population (*p* = 0.72) and toxicity was limited to elevation of lymphocytes in the combination immunotherapy group (*p* = 0.02). Exploratory baseline circulating tumor DNA (ctDNA) sequencing revealed increased survival for patients with *KRAS* wildtype tumors in both the combination immunotherapy (*p* = 0.001) and chemotherapy (*p* = 0.004) groups. These data support the utility of ctDNA analysis in PDAC and the prognostic value of ctDNA-based *KRAS* mutation status.

## Abstract (web summary)

Metastatic pancreatic ductal adenocarcinoma (mPDAC) has limited therapeutic options and is associated with a poor prognosis. Here the authors report the results of a randomized phase II trial showing that combining checkpoint inhibitors (durvalumab and tremelimumab) with chemotherapy (gemcitabine and nab-paclitaxel) does not improve survival compared to chemotherapy alone in patients with mPDAC.

## Introduction

The 5-year survival rate for patients diagnosed with metastatic pancreatic ductal adenocarcinoma (mPDAC) is 3% and remains among the lowest of all common cancer diagnoses^1^. Disease aggressiveness and delays in detection contribute to the poor prognosis associated with mPDAC, and these challenges are further compounded by the limited therapeutic options available for patients. Advances in systemic therapy over the past decade have been limited and include the adoption of FOLFIRINOX^2^ or gemcitabine and nab-paclitaxel^3^ as first line treatment options, though these therapies were shown to confer a median overall survival of only 11.1 and 8.5 months, respectively.

Immune checkpoint inhibitors (ICI) have demonstrated significant efficacy in multiple solid tumor types, but effectiveness of ICIs as single agent therapy in mPDAC remains limited^4–6^. Possible explanations for the immune resistance noted in pancreatic cancer include tumour microenvironment factors, such as the presence of cancer associated fibroblasts (CAFs), which may have an immunosuppressive effect^7^. As certain chemotherapeutic agents such as nab-paclitaxel have been shown to negate the immunosuppressive effects of CAFs and the desmoplastic stroma in which they reside^8–10^, combinatory first line treatment that includes both chemotherapy and ICI represents a promising and previously unexplored treatment strategy in mPDAC. In addition, there exists an important need to generate correlative and exploratory sequencing-based data to identify predictive biomarkers of immunotherapy response. Circulating tumor DNA (ctDNA) sequencing represents a rapid and scalable method of tumor genomics profiling. As required input consists only of patient plasma samples, ctDNA sequencing is particularly advantageous for cancers that require challenging biopsy procedures that typically limit tumor DNA yield for downstream tissue-based sequencing, as is the case for mPDAC.

Here, we present results of a multi-center, randomized, phase II trial (PA.7; NCT02879318) that aimed to assess the safety and efficacy of combination chemotherapy/ICI in mPDAC by comparing gemcitabine, nab-paclitaxel plus durvalumab (PD-L1 inhibitor) and tremelimumab (CTLA-4 inhibitor) versus gemcitabine with nab-paclitaxel alone. Durvalumab and tremelimumab are monoclonal antibody (mAb) ICIs that act through inhibition of programmed death-ligand 1 (PD-L1) and CTLA-4, respectively, and their combination has been shown to be safe in a phase Ib trial^11^. As part of an exploratory objective of the trial, we perform bioinformatics analysis of baseline ctDNA sequencing to assess the utility of incorporating plasma sequencing as a correlative dataset in prospective mPDAC trials.

## Results

### Chemotherapy combined with dual ICI does not confer a significant increase in survival among an unselected population of patients with mPDAC

**Fig. 1:**
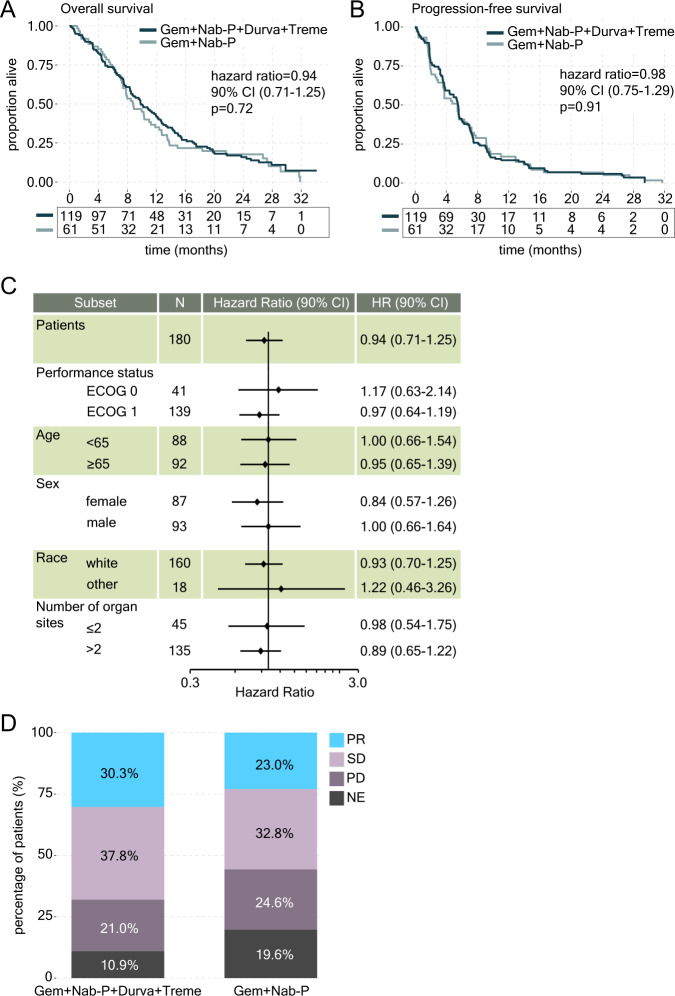
Combination dual checkpoint inhibition and chemotherapy was not associated with OS, PFS nor ORR in an unselected population of patients with metastatic PDAC. **A** Kaplan-Meier curve comparing overall survival (OS) between treatment arms. **B** Kaplan-Meier curve comparing progression-free survival (PFS) between treatment arms. **C** Forest plot showing results of subgroup analysis based on OS. Measure of center for error bars represents mean values. **D** Bar plot comparing differences in objective response rate (ORR) between treatment arms. Hazard ratio and confidence intervals (CIs) based on stratified Cox models are shown along with log-rank *p* values, and statistical tests were two-sided. Source data are provided as a source data file.

Patients with mPDAC (*n* = 180) were randomized to two treatment arms: gemcitabine, nab-paclitaxel, durvalumab and tremelimumab (hereafter referred to as ‘chemo+ICI’; *n* = 119) or gemcitabine and nab-paclitaxel (‘chemo’; *n* = 61; Supplementary Fig. 1). Treatment was initiated in all patients in the chemo+ICI arm and 58/61 (95.1%) patients in the chemo arm. Baseline characteristics were well balanced between arms (Table 1). Overall survival (OS) was compared between treatment arms per the primary endpoint of the trial. With a median follow-up of 28.5 months, there was no significant difference in OS between chemo+ICI (median OS = 9.8 months) and chemo arms (median OS = 8.8 months; hazard ratio (HR) 0.94 with 90% confidence interval [CI] 0.71–1.25; *p* = 0.72; Fig. 1A). Progression-free survival (PFS), one of the secondary endpoints of the trial, was also not significantly different between treatment arms (median PFS = 5.5 versus 5.4 months in chemo+ICI and chemo arms, respectively; HR 0.98 with 90% CI 0.75–1.29, *p* = 0.91; Fig. 1B).

**Table 1:** Baseline characteristics of intention-to-treat study population

Characteristics	Gem+Nab-P+Durva+Treme (*n* = 119)	Gem+Nab-P (*n* = 61)
**Age - years**		
Median (range)	64 (29–81)	65 (42–84)
**Sex -** ***n*** **(%)**		
Male	67 (56.3)	26 (42.6)
Female	52 (43.7)	35 (57.4)
**Race -** ***n*** **(%)**		
White	105 (88.2)	55 (90.2)
Asian	10 (8.4)	6 (9.8)
Other	4 (3.4)	0 (0)
**ECOG Performance Status -** ***n*** **(%)**		
0	27 (22.7)	14 (23.0)
1	92 (77.3)	47 (77.0)
**Prior Adjuvant Chemotherapy - n (%)**		
Yes	12 (10.1)	7 (11.5)
No	107 (89.9)	54 (88.5)

*Gem+Nab-P+Durva+Treme* Gemcitabine, nab-paclitaxel, durvalumab and tremelimumab, *Gem+Nab-P* gemcitabine and nab-paclitaxel, ECOG Eastern Cooperative Oncology Group.

Analyses in the groups defined by performance status, age, sex, race or number of organ sites with metastases revealed no significant difference in survival between treatment groups (Fig. 1C). Overall response rate (ORR), another secondary endpoint of the trial, was not significantly different between treatment groups (30.3% versus 23.0% in chemo+ICI and chemo arms, respectively; odds ratio=1.49, 90% CI: 0.81-2.72, *p* = 0.28; Fig. 1D). Disease control rate (DCR) was 70.6% (84/119) in the chemo+ICI arm vs. 57.4% (35/61) in the chemo arm (odds ratio = 1.69, 90% CI: 0.99–2.89; *p* = 0.096). Significant differences between treatment arms in grade 3 or greater laboratory abnormalities (Supplementary Table 1) or adverse events (including immune-related events; Table 2) occurring in at least 5% of either study arm were limited to elevation in lymphocytes (38% in the chemo+ICI arm versus 20% in the chemo arm; *p* = 0.02). Among patients in the chemo+ICI arm, there were three grade 4 events that were considered at least possibly related to the immunotherapy treatment, including one of each of hypertension, sepsis and pneumonitis. Also among patients in the chemo+ICI arm were two grade 5 events at least possibly related to immunotherapy treatment, including one event of colonic perforation and one event of entercolitis. Two grade 5 events that were at least possibly treatment related were noted in the chemo arm. Addition of ICI to chemo did not result in significantly more patients with deterioration in physical function or global health status at 8 weeks or 16 weeks (Supplementary Table 2). Overall, these data indicate that the combination of gemcitabine, nab-paclitaxel and dual ICI does not offer significant clinical benefit over chemotherapy alone in an unselected population of patients with mPDAC.

**Table 2:** List of grade ≥ 3 adverse events on treatment

Grade ≥ 3 AE (>5% frequency)	Gem+Nab-P+Durva+Treme (*n* = 119)	Gem+Nab-P (*n* = 58)
Any grade ≥3 AE	100 (84)	44 (76)
Fatigue	24 (20)	12 (21)
Thromboembolic event	16 (15)	7 (12)
Sepsis	13 (11)	7 (12)
Peripheral sensory neuropathy	13 (11)	7 (12)
Diarrhea	6 (5)	6 (10)
Abdominal pain	6 (5)	6 (10)
Febrile neutropenia	7 (6)	4 (7)
Vomiting	7 (6)	2 (3)
Edema limbs	5 (4)	3 (5)
Bile duct stenosis	7 (6)	0 (0)
Biliary tract infections	11 (9)	3 (5)
Lung infection	6 (5)	4 (7)
Generalized muscle weakness	1 (1)	5 (9)
Renal calculi	2 (2)	3 (5)
Dyspnea	8 (7)	4 (7)
Rash maculo-papular	7 (6)	2 (3)
Hypertension	3 (3)	4 (7)

Adverse events occurring in at least 5% of either study arm are listed. *AE* adverse event, *Gem+Nab-P+Durva+Treme* Gemcitabine, nab-paclitaxel, durvalumab and tremelimumab, *Gem+Nab-P*, gemcitabine and nab-paclitaxel.

### ctDNA-based somatic mutation landscape in mPDAC

**Fig. 2:**
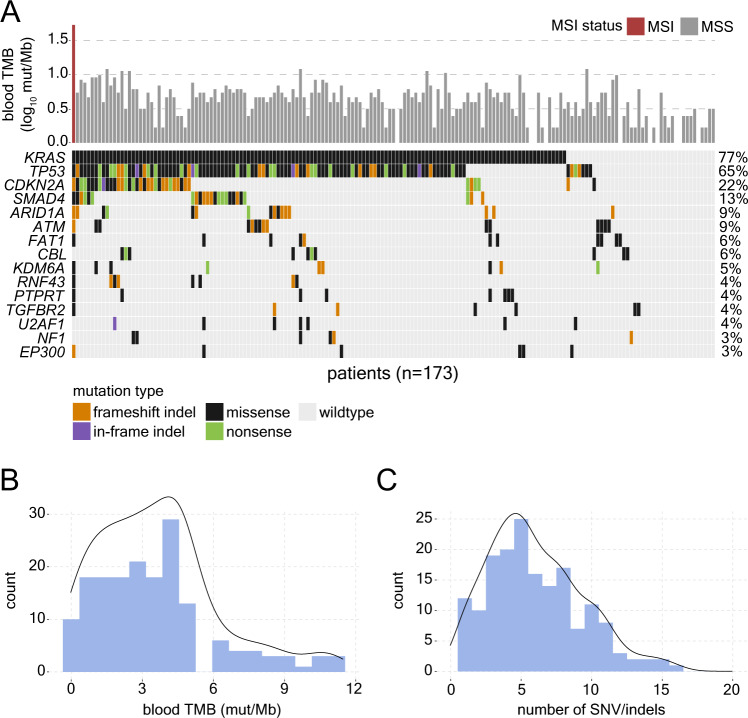
Somatic mutation landscape of mPDAC detected using liquid biopsy (ctDNA) sequencing at baseline. **A** Oncoprint depicting most frequently mutated genes detected by baseline ctDNA sequencing in the cohort of 173 patients with mPDAC. Upper bars indicate blood tumor mutation burden (TMB; mut/Mb) levels and are colored based on microsatellite instability (red) or stable (grey) status. **B** Histogram with overlaid density curve showing distribution of blood tumor mutational burden levels across the cohort. **C** Histogram with overlaid density curve showing distribution of the number of SNV/indels detected by ctDNA sequencing. Source data are provided as a source data file.

Exploratory baseline ctDNA sequencing was performed for 174 patients with available samples using the PredicineATLAS^TM^ assay, encompassing mutations across 600 genes (total of 2.4 Mb) and providing blood tumor mutation burden (bTMB) scores (mutations/Mb) and microsatellite instability status for each patient. ctDNA sequencing was successful in 173/174 (99.4%) of cases, with one sample failing quality control analysis. 172 patients were microsatellite stable while one patient showed microsatellite instability (bTMB = 52.9 mut/Mb). As a continuation of the exploratory objective of the study, we next sought to investigate genes that were frequent targets of somatic mutation (SNV/indels) across the cohort. ctDNA sequencing of baseline plasma samples detected one or more loss-of-function (frameshift/in-frame indel, nonsense or missense) somatic variants in 170/173 (98.3%) patient samples (median 5, min 1, max 74 variants). Germline mutations were also derived from the ctDNA sequencing data, and one or more loss-of-function germline variants were detected in 172/173 (99.4%) patient samples (median 5, min 1, max 13 variants). The top most frequently somatic mutated genes were *KRAS* (77% of patients) and *TP53* (65%), followed by *CDKN2A* (22%) and *SMAD4* (13%; Fig. 2A). bTMB levels were found to follow a right-skew distribution (min bTMB = 0, median = 3.7, max = 11.2 mut/Mb; Fig. 2B), while the number of SNV/indels detected in each patient followed a similar distribution (Fig. 2C). Taken together, these data support the utility of ctDNA sequencing to profile the somatic mutation landscape of patients with mPDAC.

### *KRAS* mutation status is a prognostic marker that is independent of treatment

**Fig. 3:**
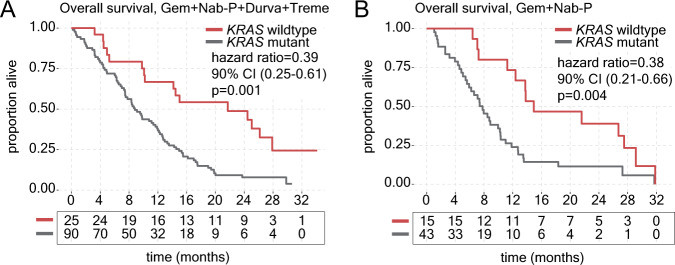
ctDNA-based *KRAS* mutation status is highly prognostic in patients with mPDAC. **A** Kaplan-Meier curve comparing overall survival (OS) between patients with *KRAS* wildtype (red) versus mutant (grey) tumors in the gemcitabine, nab-paclitaxel, durvalumab and tremelimumab (Gem+Nab-P+Durva+Treme) treatment group. **B** Kaplan-Meier curve comparing OS between patients with *KRAS* wildtype versus mutant tumors in the gemcitabine, nab-paclitaxel (Gem+Nab-P) treatment group. Hazard ratio and confidence intervals (CIs) based on stratified Cox models are shown along with log-rank *p* values, and statistical tests were two-sided. Source data are provided as a source data file.

Previous studies have demonstrated that *KRAS* wildtype tumors, which account for approximately 10-15% of PDAC^12–14^, appear to be a distinct molecularly entity from *KRAS* mutant tumors and bear alternative driver events such as *NRG1* gene fusions^15–17^. As an additional analysis towards the exploratory objective of the study, we investigated the relationship between oncogenic *KRAS* mutation and immunotherapy response. *KRAS* wildtype tumors accounted for 40/173 (23%) PA.7 samples, and *KRAS* wildtype status did not show significant overlap with germline *ATM* mutation (four of 40 (10%) patients with *KRAS* wildtype tumors had germline *ATM* mutation; *p* = 0.77). While *KRAS* mutation status was not predictive of response to combination immunotherapy (p-interaction=0.95), patients with *KRAS* wildtype tumors showed significantly improved OS in both the chemo+ICI (median OS = 21.7 months versus 8.8 months in *KRAS* wildtype versus mutant tumors, respectively; HR = 0.39, 90% CI: 0.25–0.61; *p* = 0.001; Fig. 3A) and chemo (median OS = 14.9 months versus 7.8 months in *KRAS* wildtype versus mutant tumors, respectively; HR = 0.38, 90% CI: 0.21-0.66; *p* = 0.004; Fig. 3B) arms. *KRAS* wildtype status was also associated with increased PFS in both chemo+ICI (median PFS = 10.3 months versus 4.9 months in *KRAS* wildtype versus mutant tumors, respectively; HR = 0.52, 90% CI: 0.35–0.77; *p* = 0.007) and chemo (median PFS = 9.1 months versus 3.8 months in *KRAS* wildtype versus mutant tumors, respectively; HR = 0.48, 90% CI: 0.29–0.81; *p* = 0.02) arms. Finally, *KRAS* mutation status was also associated with increased OS when patients from both treatment arms were combined (HR = 0.40, 90% CI: 0.28–0.56; *p* < 0.0001; Supplementary Fig. 2).

The frequency of *KRAS* wildtype status in the PA.7 cohort (23%) was higher than expected when compared to previous studies of mPDAC (10–15%^12–14^). It is possible that a subset of samples could have *KRAS* mutations not picked up by ctDNA analysis due to lower tumor shedding, as measured by circulating tumor fraction (CTF), the percentage of cell-free DNA that is derived from tumor cells in a blood sample. As low CTF has been associated with improved prognosis in other cancer types such as small cell lung cancer^18^, such samples could artificially inflate survival patterns of the *KRAS* wildtype group. To examine this possibility, we first compared CTF values between *KRAS* wildtype and mutant groups and noted significantly lower CTF values among the *KRAS* wildtype group (*p* = 6.6e−7). We hypothesized that there existed a conservative CTF threshold by which a subset of the cohort could be filtered such that *KRAS* wildtype frequency would approach the expected value of approximately 10%. By testing increasingly conservative CTF thresholds, we arrived at CTF = 1.68% as a threshold by which to exclude possible low-shedding samples, as the rate of *KRAS* wildtype status reached 12.7% at this threshold (Supplementary Fig. 3A). Repeating the survival analysis using only samples that met this CTF threshold (*n* = 134), *KRAS* mutation status remained prognostic in the PA.7 cohort, with patients bearing *KRAS* wildtype tumors showing increased survival (*n* = 17; median OS = 21.6 months) compared to patients with *KRAS* mutant tumors (*n* = 117; median OS = 8.7 months; HR = 0.45, 90% CI:0.27–0.76; *p* = 0.0092; Supplementary Fig. 3B) and thus opposing the possibility that patients with low-shedding tumors bias survival patterns of the *KRAS* wildtype group. Overall, these data highlight the prognostic value of ctDNA-based *KRAS* mutation status in patients with metastatic PDAC treated with gemcitabine and nab-paclitaxel-based therapies.

## Discussion

Pancreatic cancer remains a common and deadly disease for which there is critical need for improved therapeutic options. In this study, the combination of dual ICI (durvalumab plus tremelimumab) and chemotherapy (gemcitabine and nab-paclitaxel) did not yield an increased median overall survival compared to chemotherapy alone in an unselected population of patients with mPDAC (9.8 versus 8.8 months), though there was a trend towards increased disease control rate (70.6% versus 57.4%). Previous literature describing correlatives of immunotherapy response in other cancer types may offer insight into why combination chemotherapy and immunotherapy failed to achieve benefit in the PA.7 cohort of patients with mPDAC. Pan-cancer studies have revealed association between immune cell signatures and immunotherapy response^19,20^, though PDAC is known to promote an immunosuppressive microenvironment through formation of dense stromal desmoplasia^21,22^. While concurrent administration of gemcitabine plus nab-paclitaxel was poised to improve immunotherapy drug access to tumor cells through structural disruption/remodelling of the PDAC tumor microenvironment^9,10^, negative results of the PA.7 trial indicate that stromal disruption, or attempts of stromal disruption using gemcitabine plus nab-paclitaxel, was not sufficient to increase immunotherapy efficacy in the overall patient population.

The utility of ctDNA analysis in pancreatic cancer has remained questionable due to challenges involving low sensitivity^23^. Our ctDNA analysis of baseline plasma samples using the PredicineATLAS^TM^ assay demonstrated a high rate of success, with ctDNA sequencing completed for 173/174 patients, and one or more loss-of-function variants detected in 170/173 patients. By assessing the overall somatic mutation landscape across samples, our study was able to support the utility of baseline ctDNA sequencing in capturing the spectrum of mutation events in patients with mPDAC.

Our group and others have previously reported somatic fusion events in the *NRG1* gene that are uniquely found in *KRAS* wildtype PDAC and predict response to the ERBB inhibitor afatinib^15,16,24^. To date, investigation regarding the prognostic significance of *KRAS* mutation status remains limited and is likely challenged by the relatively rare occurrence of *KRAS* wildtype PDAC. Across several previous studies of tissue-based mutation profiling in mixed early-stage and metastatic cohorts, patients with *KRAS* wildtype tumors were shown to have improved survival compared to patients with *KRAS* mutant tumors, suggesting a prognostic effect of *KRAS* mutation status^14,25–28^. Here, we provide demonstration of the prognostic capability of ctDNA-based *KRAS* mutation status in mPDAC using a large cohort consisting solely of patients with metastatic disease treated with gemcitabine and nab-paclitaxel-based therapy. While *KRAS* wildtype status in the PA.7 cohort was higher than expected and potentially confounded by a subset of patients with tumors that shed lower levels of cell-free DNA into the bloodstream, we were able to verify the prognostic value of *KRAS* mutation status when the cohort was conservatively filtered to exclude patients with lower CTF values. While *KRAS* mutation status was not found to be predictive of response to combination immunotherapy, prognostic significance was noted within both treatment groups as well as the combined cohort. Considering emerging studies regarding the enrichment of actionable fusion events in *KRAS* wildtype PDAC, our results further support the usage *KRAS* mutation status testing as an early clinical strategy for patients newly diagnosed with metastatic PDAC.

It should be noted that these biomarker studies were retrospective and exploratory in nature.

Further analysis of both ctDNA data as well as tumor profiling is underway to assess for potential predictive markers of immunotherapy sensitivity. As we move forward with the next generation of PDAC trials, it will be important to move away from generic pancreatic cancer trials and move towards trials that focus on known actionable subtypes, a list that is rapidly expanding and includes *BRCA/PALB2* mutations^29,30^, mismatch repair deficiency^31^ (MMRd), *NRTK* fusions^32^ and *NRG1* fusions^15^. In addition, other actionable subtypes currently being explored include high TMB, *KRAS* wildtype status and germline *ATM* mutations, as well as homologous recombination deficiency (HRD), other fusion drivers (FGFR2), *HER2* overexpression, and *BRAF* mutations. The next generation of trials in PDAC should aim to validate the predictive role of these subtypes to define their utility in treatment selection, specifically sensitivity to immunotherapy, targeted therapy, and chemotherapy. In regards to chemotherapy sensitivity, the ongoing PASS-01 trial (NCT04469556), whereby PDAC patients undergo comprehensive genomic analysis and are randomized to FOLFINOX vs. gemcitabine and nab-paclitaxel, will inform the utility of these subtypes in chemotherapy selection. The correlative ctDNA-based analysis performed as part of the PA.7 trial suggests that future trials should incorporate ctDNA analyses as one of the planned correlative studies.

In conclusion, the CCTG PA.7 trial did not demonstrate a benefit from adding durvalumab and tremelimumab to gemcitabine and nab-paclitaxel as a first line therapy in an unselected population of patients with mPDAC. However, *KRAS* mutation status showed correlation with survival outcome across the PA.7 cohort, with patients bearing *KRAS* wildtype tumors showing increased survival independent of treatment type. Importantly, this study demonstrates the potential utility of next generation ctDNA platforms in PDAC, and consideration should be given towards including ctDNA analysis as a correlative study for ongoing and future PDAC trials. Further studies are needed both to improve our understanding of the role of immunotherapy in mPDAC and also to better elucidate the mechanisms of immunotherapy resistance and sensitivity biomarkers.

## Methods

### Trial design and patient enrollment

PA.7 received institutional ethics review board approval and was carried out per the Declaration of Helsinki and International Ethical Guidelines for Biomedical Research Involving Human Subjects (NCT02879318). The study protocol was approved by REB of participating centers, the Ontario Cancer Research Ethics Board and UBC BC Cancer Research Ethics Board. Patients provided written informed consent. PA.7 was a randomized phase II clinical trial conducted within 28 centres across Canada. After a safety run-in which included 11 patients, 180 patients diagnosed with metastatic pancreatic ductal adenocarcinoma (mPDAC) were enrolled in the study. Trial inclusion criteria were: patients must have histologically or cytologically confirmed PDAC which is metastatic, must have presence of measurable or evaluable disease as defined by Response Evaluation Criteria in Solid Tumors (RECIST 1.1), must be considered suitable candidates and able to receive first line chemotherapy for metastatic disease with gemcitabine and nab-paclitaxel, must consent to provision of a formalin-fixed paraffin block of tumor tissue and samples of blood, serum and plasma, must be at least 18 years of age with an ECOG performance status of zero or one with a life expectancy of at least 12 weeks, must have not received prior treatment for metastatic disease, must have adequate normal organ and marrow function, have an imaging investigation including CT/MRI of chest/abdomen/pelvis to document sites of disease within 28 days prior to randomization, must be able to complete quality of life questionnaires and must be accessible for treatment and follow-up. Exclusion criteria included: history of other malignancies, previous treatment with PD1 or PD-L1 inhibitor, history of primary immunodeficiency, active or prior documented autoimmune or inflammatory disorders and active or uncontrolled intercurrent illness. Patients were randomized to receive gemcitabine (1000 mg/m2 D1, 8, 15), nab-paclitaxel (125 mg/m2 D1, 8, 15), durvalumab (1500 mg D1 q 28 days) and tremelimumab (75 mg D1 for first 4 cycles) versus gemcitabine and nab-paclitaxel alone in a 2:1 ratio (respectively). Randomization was dynamically balanced by ECOG performance status (0 vs 1) and receipt of prior adjuvant therapy (yes versus no) using the method of minimization. Randomization was performed centrally by the Canadian Cancer Trials Group (CCTG) central office. Overall survival (OS) was the primary endpoint. Secondary endpoints included progression free survival (PFS), safety, overall response rate (ORR) and quality of life assessed by EORTC QLQ-C30. Accrual of patients started on August 22, 2016. Randomized Phase II component of this study was opened on April 10, 2017 after the review of the results from 11 safety run-in patients and closed on July 28, 2018 after the final patient was randomized. The CCTG Data and Safety Monitoring Committee regularly evaluated the conduct and safety of the study.

### cfDNA extraction, library preparation, probe capture and sequencing

Circulating cell-free DNA was extracted by the QIAamp circulating nucleic acid kit from plasma samples. Quantity and quality of the purified cfDNA were checked using Qubit fluorimeter and Bioanalyzer 2100. For samples with severe genomic contamination from peripheral blood cells, a bead-based size selection was performed to remove large genomic fragments. Five to 30 ng of extracted cfDNA were subjected for library construction including end-repair dA-tailing and adapter ligation. Ligated library fragments with appropriate adapters were amplified via PCR. The amplified DNA libraries were then further checked using Bioanalyzer 2100 and samples with sufficient yield are proceeded to hybrid capture.

The 600-gene PredicineATLAS^TM^ panel with Biotin labelled DNA probes was used for target enrichment. In brief, the library was hybridized overnight with Predicine NGS panel and paramagnetic beads. The unbound fragments were washed away, and the enriched fragments were amplified via PCR amplifications. Similarly as library preparation, the purified product was checked on Bioanalyzer 2100 and then loaded into Illumina NovaSeq 6000 for NGS sequencing with paired-end 2x150bp sequencing kits.

### Analyses of NGS data from cfDNA

NGS Data was analyzed using Predicine DeepSea NGS analysis pipeline, which starts from the raw sequencing data (BCL files) and outputs the final mutation calls. Briefly, the pipeline first performs adapter trimming, barcode checking, and correction. Cleaned paired FASTQ files are aligned to human reference genome build hg19 using the BWA alignment tool. Consensus BAM files are then derived by merging paired-end reads originated from the same molecules (based on mapping location and unique molecular identifiers) as single-strand fragments. Single-strand fragments from the same double-strand DNA molecules were further merged as double-stranded. By using error suppression method described by Newman and colleagues^33^, both sequencing and PCR errors were mostly corrected during this process.

Candidate variants were called by comparing with local variant background (defined based on plasma samples from health donors and historical data). Variants were further filtered by log-odds (LOD) threshold^34^, base and mapping quality thresholds, repeat regions and other quality metrics.

### bTMB score estimation

Blood-based tumor mutational burden (bTMB) was defined as the number of somatic coding single nucleotide variants (SNVs) including synonymous and nonsynonymous variants within panel target regions. The bTMB score was then normalized by the total effective targeted panel size within the coding region^35^. As there were no matched normal samples, such as peripheral blood mononuclear cells (PBMCs), available for germline variant filtering, germline variants were inferred based on variant annotation, variant allele frequency, and other variant information such as variant copy number status. Variants in common clonal haematopoietic mutations of indeterminate potential (CHIP) genes (*DNMT3A*, *TET2*, *ASXL1* and *JAK2*) were excluded in bTMB estimation.

### Circulating tumor fraction estimation

Circulating tumor fractions were estimated based on the allele fractions of autosomal somatic mutations as described previously^36^. Briefly, the mutant allele fraction (MAF) and ctDNA fraction are related as MAF = (ctDNA*1)/[(1-ctDNA)*2 + ctDNA *1], and so ctDNA = 2/((1 / MAF) + 1). Somatic mutations in genes with a detectable copy number change were omitted from circulating tumor fraction estimation.

### ctDNA analysis

For somatic and germline variants, frameshift and in-frame insertion/deletions (indels), missense and nonsense mutations were included in our analysis. For the secondary survival analysis of *KRAS* wildtype samples, CTF was used as a metric by which to conservatively filter samples. To determine the CTF threshold by which to exclude samples, we iteratively tested increasing CTF thresholds. At each iteration, samples with CTF values less than the threshold were excluded and the frequency of *KRAS* wildtype status was recalculated. A *KRAS* wildtype frequency of approximately 10%, while retaining a large portion of patients, was reached at CTF = 1.68%, and this value was therefore chosen as the threshold by which to exclude samples in the secondary *KRAS* wildtype survival analysis as the rate of *KRAS* wildtype status was more similar to that of previous studies^12–14^.

### Statistics

The primary end point OS was defined as the time from the date of randomization to the date of death from any cause. The secondary end point PFS was defined as the time from the date of randomization to the first date when the disease progression was objectively documented or the date of death from any cause and ORR as the proportion of randomized patients with a documented complete response or partial response. This study was designed to detect, with an 80% power and a 2-sided 10% level, a HR of 0.65 between two treatment arms, which corresponded to an increase of the median OS from 8.5 months for the chemo arm to 13.1 months for the chemo+ICI arm and required observation of 150 deaths before the final analysis.

OS and PFS were summarized by Kaplan-Meier method and compared by a log-rank test stratified by ECOG performance status and prior adjuvant therapy. Stratified Cox models were used to calculate hazard ratios (HRs) and 90% confidence intervals (CIs). ORR between treatment groups were compared by a Cochran-Mantel-Haenszel test stratified by ECOG Performance Status and prior adjuvant therapy. Fisher’s exact test was used to compare rates of adverse events between treatment arms as well as the QOL primary endpoints prespecified as proportions of patients who had deterioration (defined as a change score from baseline which is –10 points or lower) in physical function and Global Health Status at 8 weeks and 16 weeks after the randomization. Two-tailed Wilcoxon mean rank-sum test was used to compare CTF values between *KRAS* wildtype and mutant groups. Primary analyses included all patients randomized, while ctDNA analysis included patients with successful ctDNA sample sequencing. SAS statistical software (version 9.0; SAS Institute, Inc) and R v3.6.3 were used for analyses.

### Reporting summary

Further information on research design is available in the Nature Research Reporting Summary linked to this article.

## Supplementary information

Supplementary Information

Reporting Summary

## Data Availability

The Canadian Cancer Trials Group (CCTG) has a data sharing policy, the details of which are available at https://www.ctg.queensu.ca/docs/public/policies/DataSharingandAccessPolicy.pdf. Data collected from this study apart from raw sequencing files, including variant calls and related clinical information, will be made available to interested researchers while respecting patient privacy. Raw sequencing data is not available as some aspects of the bioinformatics pipeline are proprietary, however individual queries can be submitted and discussed with CCTG and Predicine. CCTG has an established request procedure and interested investigators should submit a brief proposal using the Request for Data Proposal Form available at https://www.ctg.queensu.ca/public/policies (to be submitted by e-mail to: datasharing@ctg.queensu.ca). Data are available under restricted access, and the policy is described at: https://www.ctg.queensu.ca/public/policies. Upon approval, de-identified individual participant data and relevant study documents (protocol and statistical analysis plan) will be made available in a reasonable timeframe, or summary data of a requested analysis can be performed in collaboration with CCTG. The remaining data are available within the Article, Supplementary Information or Source Data file. Source data are provided with this paper.

## Source data

Source Data
