# Supplementary material for: The CCTG PA.7 phase II trial of gemcitabine and nab-paclitaxel with or without durvalumab and tremelimumab as initial therapy in metastatic pancreatic ductal adenocarcinoma
Source: Nat Commun. 2022 Aug 26;13:5020. doi: 10.1038/s41467-022-32591-8 (PMC9418247; doi:10.1038/s41467-022-32591-8)
Supplement: Supplementary file 1 — Supplementary Information [file 41467_2022_32591_MOESM1_ESM.pdf]

**Grade 3/4 Laboratory Abnormality (>5%)**	**G+N+D+T; Evaluable patients=119 (%)**	**G+N; Evaluable patients=58 (%)**
Anemia	26 (22)	18 (32)
WBC	42 (36)	17 (31)
Lymphocytes*	45 (38)	11 (20)
Neutrophils	58 (49)	25 (44)
Platelets	13 (11)	9 (16)
Hyponatremia	10 (8)	7 (12)
Hypokalemia	9 (8)	2 (4)
Bilirubin	9 (8)	4 (7)
Alkaline Phosphatase	15 (13)	6 (11)
SGOT (AST)	9 (8)	2 (4)
SGPT (ALT)	10 (9)	5 (9)
Hypoalbuminemia	7 (6)	6 (11)
Amylase	7 (6)	3 (5)
Lipase	15 (13)	10 (20)

Supplementary Table 1: Laboratory abnormalities for patients on treatment. Grade 3 and 4 laboratory abnormalities are summarized for each treatment arm. Abbreviations: G+N+D+T, Gemcitabine, nab-paclitaxel, durvalumab and tremelimumab; G+N, gemcitabine and nab-paclitaxel. Asterisk indicates statistical significance (Fisher's exact test p=0.02). Statistical tests are two-sided.

		N	G+N+D+T<br>N (%)	G+N<br>N (%)	P value*
Physical function					
	Week 8	134	31 (33.3)	11 (26.8)	0.54
	Week 16	112	29 (36.7)	8 (24.2)	0.46
Global health status					
	Week 8	131	27 (29.3)	9 (23.1)	0.42
	Week 16	111	24 (30.8)	5 (15.2)	0.24

Supplementary Table 2: Proportion of patients with deterioration in quality of life. Abbreviations: G+N+D+T, Gemcitabine, nab-paclitaxel, durvalumab and tremelimumab; G+N, gemcitabine and nab-paclitaxel. *From Fisher's exact test. Statistical tests are two-sided.

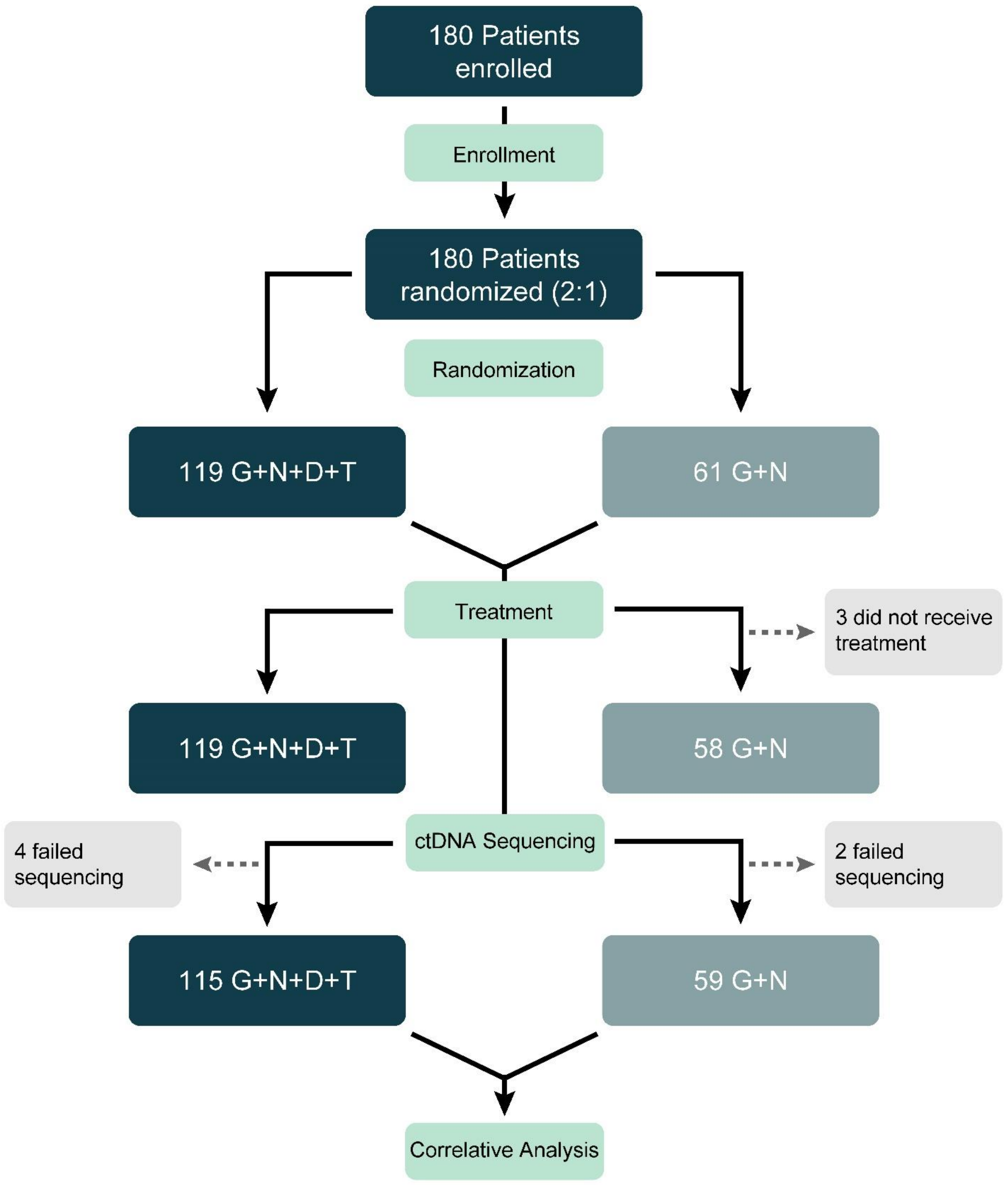

G+N+D+T=Gemcitabine/Nab-paclitaxel plus Durvalumab and Tremelimumab
G+N=Gemcitabine/Nab-paclitaxel

Supplementary Fig. 1: CONSORT diagram showing study design for the Canadian Cancer Trials Group (CCTG) PA.7 trial.

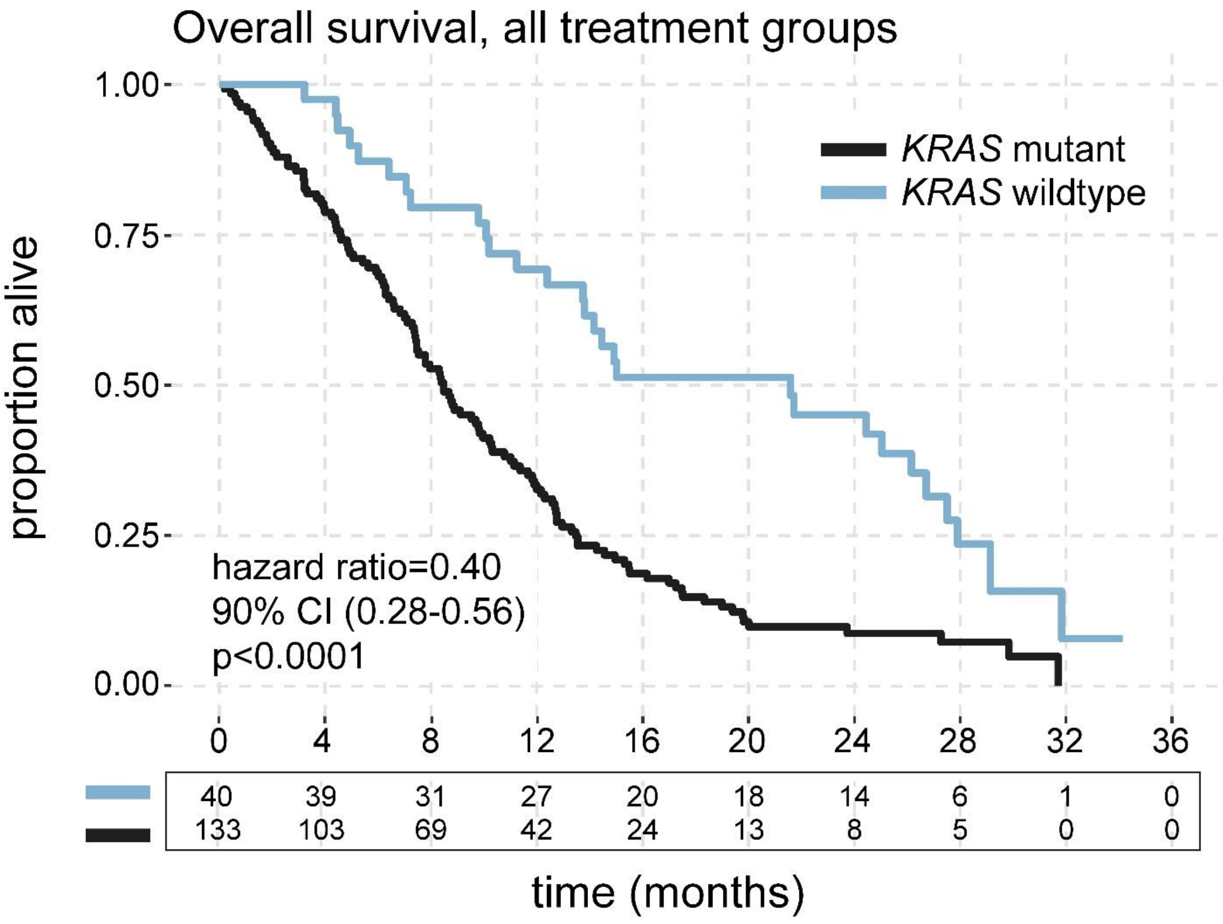

Supplementary Fig. 2: Comparison of overall survival for patients bearing oncogenic *KRAS* mutation, across all treatment arms. Kaplan-Meier curve comparing overall survival (OS) between patients with *KRAS* wildtype (blue) and mutant (black) tumors. Hazard ratio and confidence intervals (CIs) based on stratified Cox models are shown along with log-rank p values, statistical tests are two-sided and patients from both treatment arms are combined.

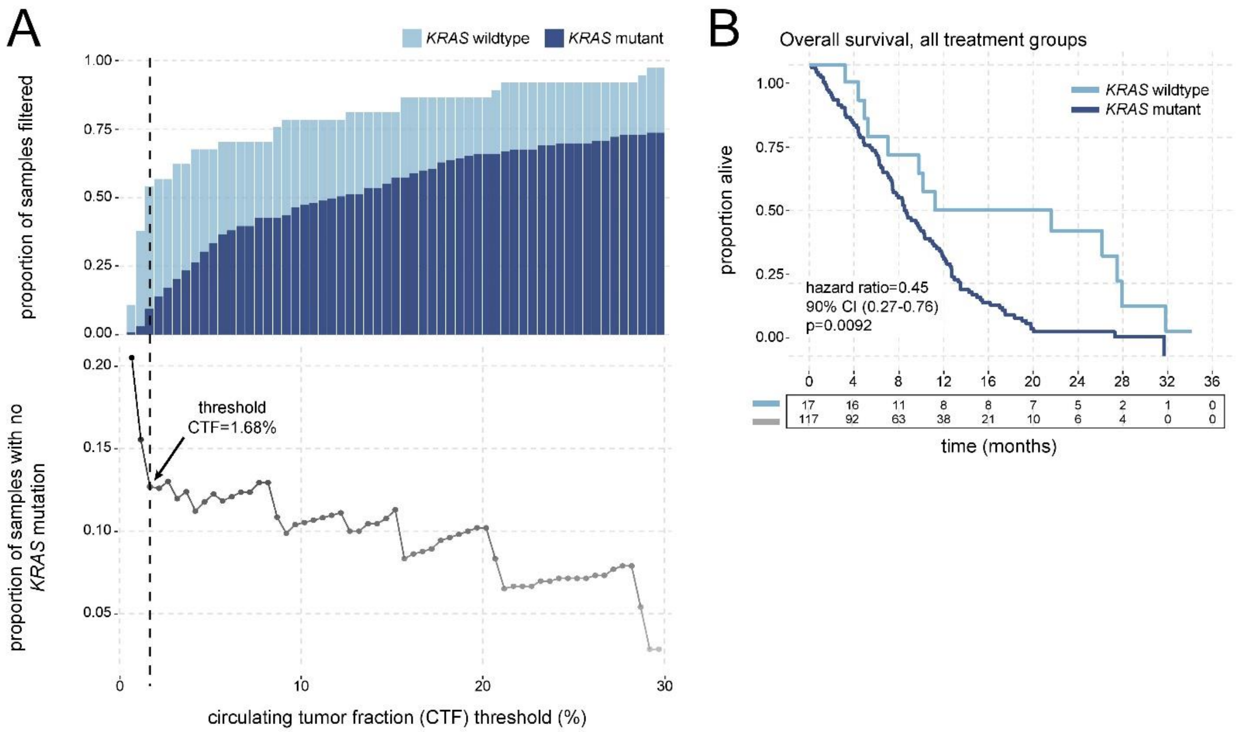

Supplementary Fig. 3: Oncogenic *KRAS* mutation conservative filtering analysis. (A) Iterative filtering of samples based on circulating tumor fraction (CTF) values. Bar plot (upper) depicts the proportion of *KRAS* mutant (dark blue) or wildtype (light blue) samples that were excluded across varying CTF values. Bars are overlaid rather than stacked. Line plot (lower) shows the overall proportion of samples with *KRAS* wildtype status when each CTF threshold was applied. A threshold CTF=1.68% was selected to conservatively filter samples, as an expected rate of *KRAS* wildtype status (12.7% of patients) was observed at this threshold (dashed vertical line). (B) Kaplan-Meier curve comparing overall survival (OS) between patients with tumors with and without oncogenic *KRAS* mutation, for patients that had CTF>1.68% (n=134). Hazard ratio and confidence intervals (CIs) based on stratified Cox models are shown along with log-rank p value, statistical tests are two-sided and patients from both treatment arms are combined.
